# Fast estimation of plant growth dynamics using deep neural networks

**DOI:** 10.1186/s13007-022-00851-9

**Published:** 2022-02-20

**Authors:** Gabriella E. C. Gall, Talmo D. Pereira, Alex Jordan, Yasmine Meroz

**Affiliations:** 1 School of Plant Sciences and Food Security, Tel Aviv University, Tel Aviv, Israel; 2 Zukunftskolleg, Department of Biology, University of Konstanz, Konstanz, Germany; 3 Centre for the Advanced Study of Collective Behaviour, Konstanz, Germany; 4 Princeton Neuroscience Institute, Princeton University, Princeton, USA; 5 Computational Neurobiology Lab, Salk Institute for Biological Studies, La Jolla, USA; 6 Department of Collective Behaviour, Max Planck Institute of Animal Behavior, Konstanz, Germany

**Keywords:** Plant movement, Tracking, SLEAP, Time-lapse imagery, Pose estimation, Deep learning, Motion capture

## Abstract

**Background:**

In recent years, there has been an increase of interest in plant behaviour as represented by growth-driven responses. These are generally classified into nastic (internally driven) and tropic (environmentally driven) movements. Nastic movements include circumnutations, a circular movement of plant organs commonly associated with search and exploration, while tropisms refer to the directed growth of plant organs toward or away from environmental stimuli, such as light and gravity. Tracking these movements is therefore fundamental for the study of plant behaviour. Convolutional neural networks, as used for human and animal pose estimation, offer an interesting avenue for plant tracking. Here we adopted the Social LEAP Estimates Animal Poses (SLEAP) framework for plant tracking. We evaluated it on time-lapse videos of cases spanning a variety of parameters, such as: (i) organ types and imaging angles (e.g., top-view crown leaves vs. side-view shoots and roots), (ii) lighting conditions (full spectrum vs. IR), (iii) plant morphologies and scales (100 μm-scale *Arabidopsis* seedlings vs. cm-scale sunflowers and beans), and (iv) movement types (circumnutations, tropisms and twining).

**Results:**

Overall, we found SLEAP to be accurate in tracking side views of shoots and roots, requiring only a low number of user-labelled frames for training. Top views of plant crowns made up of multiple leaves were found to be more challenging, due to the changing 2D morphology of leaves, and the occlusions of overlapping leaves. This required a larger number of labelled frames, and the choice of labelling “skeleton” had great impact on prediction accuracy, i.e., a more complex skeleton with fewer individuals (tracking individual plants) provided better results than a simpler skeleton with more individuals (tracking individual leaves).

**Conclusions:**

In all, these results suggest SLEAP is a robust and versatile tool for high-throughput automated tracking of plants, presenting a new avenue for research focusing on plant dynamics.

**Supplementary Information:**

The online version contains supplementary material available at 10.1186/s13007-022-00851-9.

## Introduction

Interest in the study of plant behaviour, first described by Charles Darwin himself [1], has been steadily rising in the last two decades [2–8]. Plant behaviour has been defined as an individual’s response to some event or environmental change during the course of its lifetime [5], and thus a form of phenotypic plasticity [2, 3]. As in animals, many behaviours in plants are expressed as a form of movement, however, due to the sessile nature of plants, most movements are directly linked to growth. Such growth-driven plant movements can generally be classified into nastic (internally driven) and tropic (environmentally driven) movements. Nastic movements include circumnutations, a circular movement of plant organs commonly associated with search and exploration, while tropisms refer to the directed growth of plant organs toward or away from environmental stimuli, such as light and gravity. Such growth-driven movements occur over long time scales, e.g., hours to days [2, 3], and are manifested by a continuously changing morphology, as opposed to the locomotion of animals. Accordingly, when studying plant behaviour, it may be unclear how behaviours are expressed and thus which behaviours to analyse. Most plants are decentralized organisms with a modular structure composed of repeating units of leaves, shoots and roots, each of which can respond simultaneously to different local stimuli. With growth being a slow process, and responses happening in different places at once, observing plant behaviour can be challenging. Time-lapse imaging offers a great opportunity to record dynamic changes in behaviour of plants over long periods of time across the whole plant, e.g., tracking shoot and leaf growth. Unlike static phenotyping, however, this form of data collection quickly yields sequences of hundreds or more images, making manual quantification of the morphological dynamics laborious if not intractable.

Previous studies have relied on classical image processing techniques to automate this process, such as dense optical flow [9] based on algorithms first introduced by Lucas and Kanade [10] as well as Horn and Schunk [11]. These algorithms measure movement directly rather than identifying specific plant parts across each frame. Depending on the biological question, these algorithms can be useful for extracting general motion features, but they lack the ability to track the trajectory of specific morphological features of interest. Other approaches leverage segmentation (foreground separation) as a pre-processing step to specialized feature extraction procedures. RootStem Extractor [12] defines the plant’s skeleton by drawing points along a line through the base of the plant and extrapolating along the line to find the outer edges of plant organs. Here leaves or cotyledons serve as endpoints for the skeleton, meaning that only shoots and roots of young plants can be tracked. Phytotyping^4D^ [13, 14] provides another tool that can be used to analyse plant movement. While this method allows to collect a whole range of detailed data, such as the location and height of plant parts, it requires a fairly complex setup and only allows for a maximum of five pictures per hour and is thus limited in the maximum temporal resolution of behaviours recorded.

In recent years, the development and use of machine learning algorithms, such as convolutional neural networks (CNNs) have greatly advanced the state-of-the-art in image-based data processing tasks across the fields of behaviour and neuroscience [15–19]. For motion capture applications, CNNs enable automatic estimation of the posture of an animal without the need of additional markers on the animal itself. Instead, these networks exhibit exceptional capabilities for learning the structure of natural features of the animal’s body, such as joints, from the image patterning alone [19, 20]. This suggests that approaches for markerless pose estimation may be suited for plants as well—a crucial step forward since markers placed on plants can directly influence their growth and movement, for example by locally inhibiting photosynthesis or by triggering a touch response. In addition to obviating the need for specialized physical equipment, such as markers or special imaging hardware, these approaches also do not require segmentation, which is a typically error-prone or manually laborious pre-processing step involved in most computational approaches for plant phenotyping.

Here we evaluated the suitability of an animal pose tracking framework, Social LEAP Estimates Animal Poses (SLEAP; www.sleap.ai; [20]), to the task of plant tracking. SLEAP is a deep learning-based tool developed for multi-animal pose tracking using CNNs. The program offers an easy-to-use graphical user interface (GUI) for image labelling and allows for GPU-free training and inference using Google Colab. It is highly accessible, with complete documentation and tutorials available, including datasets. Importantly, SLEAP is particularly useful for this application as it supports training customizable lightweight neural network models; these small networks function as “specialists” which are fast to train (< 1 h) and can work few (< 10–100) labelled images. This differs from most deep neural network architectures which rely on large-scale datasets (> 1000 labelled images) for training “generalist” models. As we will show, this unique feature is key to enabling efficient labelling for typical plant phenotyping projects in lab settings which typically do not exceed 1000 frames. We used time-lapse videos from five separate setups and three different types of plants (*Arabidopsis,* bean, and sunflower) differing in imaging angles (side-view and top-view), lighting conditions (full spectrum, blue and infrared light), scales (100 μm-scale and cm-scale), movement types (circumnutations, tropisms and twining) and organ types being tracked (shoots, roots and leaves). Unlike animals, plant morphology changes over the course of a time-lapse video, introducing a problem since SLEAP requires the “skeleton” (the fixed set of morphological landmarks to track) to be pre-defined during labelling. Hence, we leverage SLEAP’s high model training efficiency to explore different model configurations and approaches optimized for each dataset, to deal with this challenge and to achieve reliable tracking with few labels.

## Methods

For this study we analysed the movement of different plants from time-lapse videos which were generated for different experimental protocols (Table 1). Each experiment name includes the plant species and type of plant movement being tracked, and an acronym for convenience: *Arabidopsis* gravitropism (AG), *Arabidopsis* phototropism (AP), bean twining (BT), sunflower phototropism (SP). In the case of the sunflower shade avoidance experiments, which involve the top view of a plant crown, we adopted two different labelling definitions, where individuals are defined as either single leaves (STL) or as whole plants (STP). Table 1 details the lighting regime and viewing direction, the resulting image dimensions and video size, the number of individuals tracked simultaneously within the same frame, and lastly the number of labels per individual. The skeleton used in each experiment are illustrated in Fig. 2.

**Table 1:** Overview of videos and analyses for movement tracking using SLEAP

Experiment and acronym	Lighting	View	Video size [MB]	Dimensions [pixels]	# Frames	# Individuals	# Labels per individual
*Arabidopsis* Gravitropism (AG)	Full spectrum	Side	0.35	462 × 714	450	5	8
*Arabidopsis* Phototropism (AP)	IR and short burst of blue light	Side	1.36	1226 × 524	180	4	5
Bean Twining (BT)	Full spectrum	Side	71.8	1990 × 2792	645	1	8
Sunflower Phototropism (SP)	Blue light	Side	8.32	1080 × 720	345	5	6
Sunflower shade avoidance (STL)	Full spectrum	Top	5.7	1280 × 960	90	21	4
Sunflower shade avoidance (STP)	Full spectrum	Top	5.7	1280 × 960	90	3	19

The names of the experiments include the plant species, the type of growth-driven movements being tracked, and an acronym. Corresponding images of the plants are shown in Fig. 2, illustrating the view direction, the number of labels per plant, and the number of individuals being tracked in each frame. We analysed the sunflower shade avoidance video in two ways, tracking separate leaves (STL) and tracking separate plants (STP). An example of labelling and tracked frames for each experiment can be found in Fig. 2

### Description of plant growth and setup

#### *Arabidopsis* gravitropism (AG)

*Arabidopsis thaliana* was grown in 12/12 h day-night cycle, on half-strength MS phytagel medium, in the growth chamber at 24 °C for 72–96 h. After germination, seedlings were transferred to another Petri dish under a biological hood. Seedlings were arranged by groups of five and the roots were placed between the lid of the Petri dish and a block of fresh phytagel (same recipe as for cultivation). The Petri dish was then mounted on a rotating plate, the inclination of which is set by an Arduino microcontroller. During the experimental period, the device keeps the Petri dish vertical (seeds growing upward) for 10 h, then tilts it 90 degrees for 48 min, and then tilts it back to its initial orientation for the rest of the experiment for 8 h. White light was provided from the top only, except when taking pictures (light from side). Pictures were taken every 10 min using a Nikon D750. For this study we only used the images taken after the tilting of the plants.

#### *Arabidopsis* phototropism (AP)

*Arabidopsis* seedlings were germinated as in (a). After germination, seedlings were transferred into small 3D printed cases which left only the hypocotyl exposed to light. As for gravitropism protocol, roots were placed between a wall of the box and a fresh phytagel block. During the experiment, seedlings were left in complete darkness during the experiments and pictures were taken every 10 min using to near infra-red (peak wavelength: 940 nm) flashes. A single transient blue-light stimulation (10 s) was provided using a set of LEDs from the left side.

#### Bean twining (BT)

Beans (*Phaseolus vulgaris*) were germinated in soil at 24 °C and a 16/8 h day/night cycle. After germination they were transferred to individual (9 × 9 cm) pots with 20–20-20 N-P-K fertilizer until they reached about 30 cm in height. The plants were then transferred into the experimental setup and were each offered a hanging straw as support for twining. Pictures were taken every 2 min from the side with a Nikon D7500.

#### Sunflower phototropism (SP)

Sunflower (*Helianthus annus*) seeds were kept in a fridge (5 °C) for 12 h before peeling them and soaking them for 24 h in water. They were than germinated in vermiculite within 50 ml tubes and a 12 h day and night cycle at 24 °C. After germination small orange dots (~ 1 mm) were painted with acrylic paint along the stem of the seedlings. The seedlings were then placed in groups of 5 within black-painted tube-racks into the experimental setup, where they were subjected to unilateral blue light (with wavelength of 450 nm, and light intensity between 300–1200 μW/cm^2^ depending on location) for ~ 22 h. Pictures were taken every 4 min throughout the experiment using a D3400 Nikon camera.

#### Sunflower shade avoidance (STL & STP)

After germination of sunflower seeds as in (d), seedlings were transferred into soil within 9 × 9 cm plastic pots. The plants were then grown with a day-night cycle of 16/8 h at ~ 22 °C. We grew three plants in a row, with pots being right next to each other. Pictures were taken every 5 min from ~ 40 cm above the plants, using a webcam (Logitech c270 webcam) connected to a Raspberry Pi (Raspberry Pi 4 model B, Pi Foundation, Cambridge, UK).

### Labelling and training of videos

**Fig. 1:**
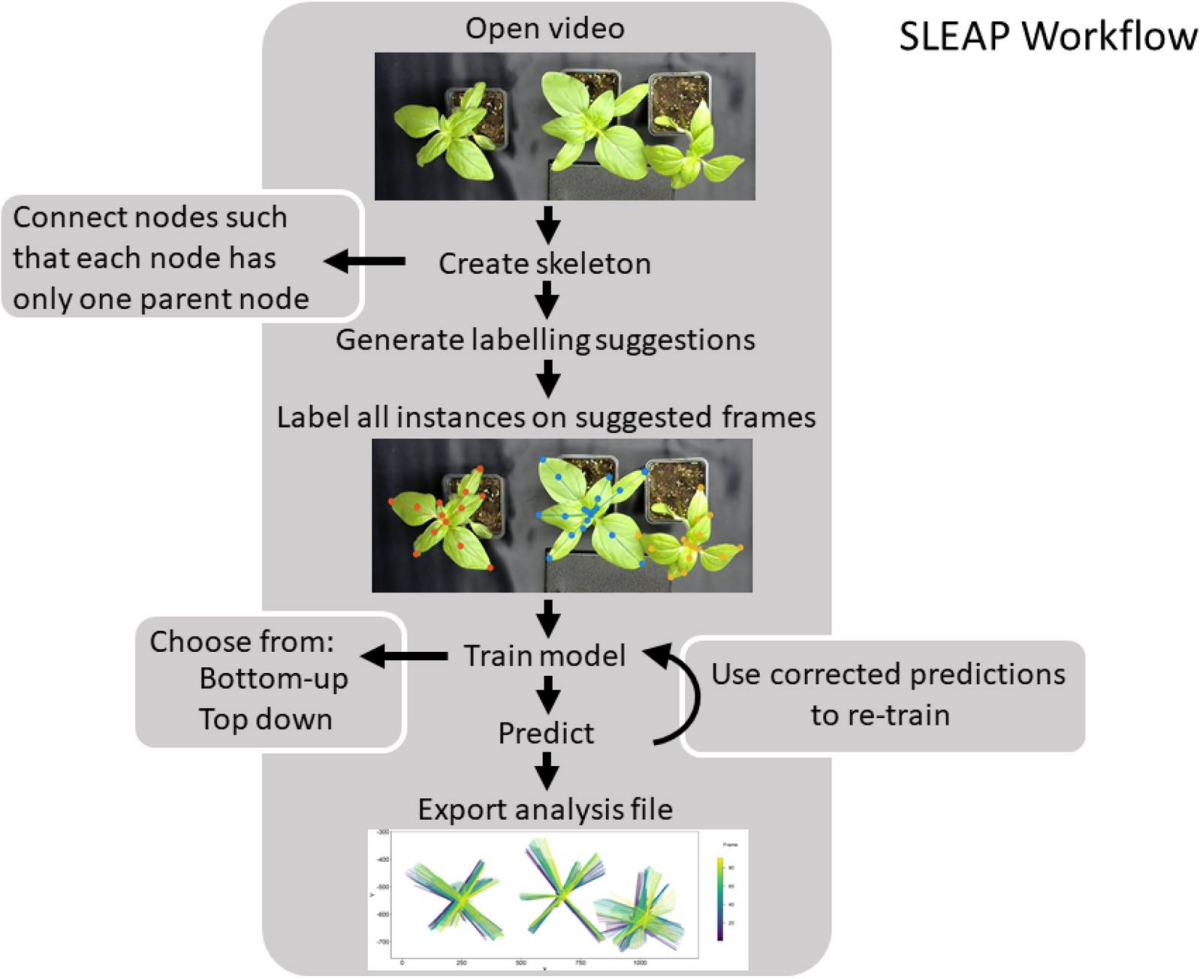
Simplified overview of the SLEAP workflow

**Fig. 2:**
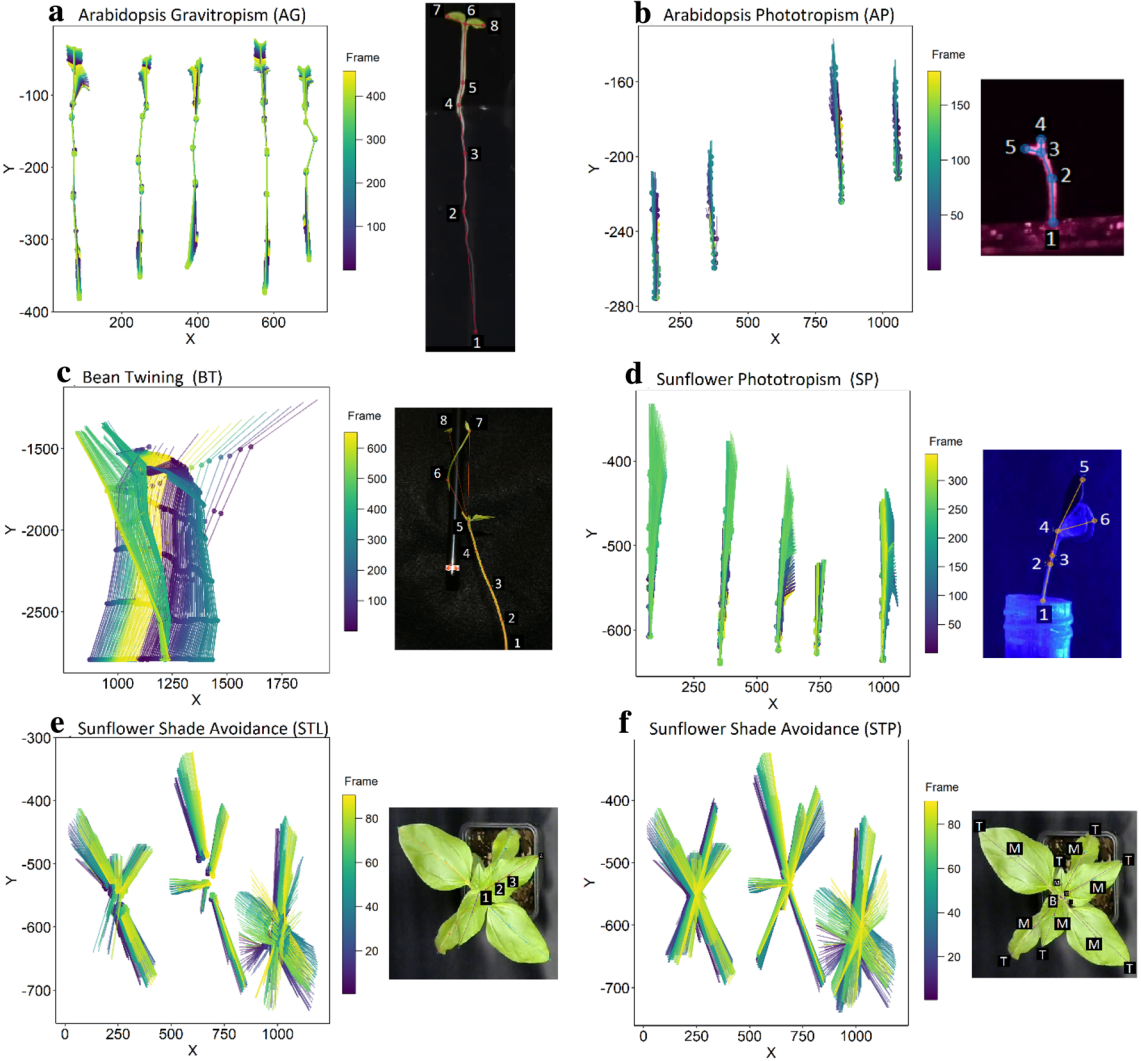
Examples for predicted movement for each video analysis across all the frames of a video using the model trained on 20 user labelled frames, as well as the skeleton used for labelling and training. **a** *Arabidopsis* Gravitropism, **b** *Arabidopsis* Phototropism, **c** Bean Twining, **d** Sunflower Phototropism, **e** Sunflower Shade Avoidance, tracking individual leaves, **f** Sunflower Shade Avoidance, tracking individual plants. See additional file for animated movement trajectories

Each video was labelled within the SLEAP (v1.1) GUI, see Fig. 1 for an overview over the SLEAP workflow and Fig. 2 for an overview of the skeleton used for each video analysis. To test the influence of the number of labelled frames on prediction accuracy, we trained the networks with 5, 10 and 20 labelled frames per video. The first five labelling frames were distributed evenly across the video in order to capture the variation of plant morphology over time due to growth. We saved the training package used for training the networks in Google Colab. All additional labels were randomly distributed across the video, and after reaching 10 and 20 labelled frames respectively we again saved the training package.

SLEAP offers two approaches to train models for identifying the posture of multiple individuals: “top-down” and bottom-up”. In the top-down mode, a network first finds the different “instances” (here: individual plants) and then estimates the location of each part (morphological landmarks belonging to the same plant). In the bottom-up approach, first all landmarks are identified and only then are they grouped into instances. While an “instance” typically refers to a single organism, SLEAP is agnostic to the definition and can be used to track repeated morphological features, such as leaves. Following preliminary testing, we chose the bottom-up approach to train our models, though which approach makes more sense depends on the model system and biological question. We employ the UNet architecture in SLEAP, configured with an encoder with 5 downsampling blocks of 2 convolutions with ReLU followed by max pooling with a stride of 2; the decoder portion of the network uses a bilinear upsampling layer (stride of 2) with 2 convolutions with ReLU until reaching an output stride of 2; the filters scale by a factor of 1.5 at each block and skip connections connect the encoder and decoder, as previously described [20]. To train the SLEAP models, we used Google Colab and code provided in the SLEAP tutorials (www.sleap.ai), whereby we recorded the time needed to train the networks. Because the bean twining video was large and resulted in out-of-memory errors, we resized it by setting the ‘input scaling’ to 0.5 in the training configurations, before training on this video.

For training, SLEAP trains models for a maximum of 200 epochs (which are rarely reached before converging), where an epoch is defined as the total number of batches that are required to perform one iteration over the training dataset, or 200, whichever is smaller, with a batch size of 4, initial learning rate of 1e-4 with the Adam optimizer as previously described [20]. The learning rate is reduced by a factor of 0.5 after the loss fails to improve by 1e-8 for 10 epochs, and training is terminated after 200 maximum epochs or 20 epochs without improvement. After training we predicted the position of each plant part using the SLEAP GUI on the 20 user labelled frames (for the accuracy analyses) as well as the whole video. For this we set the maximum number of individuals to be identified in each video (see Table 1 for details on the number of individuals per video analysis). We used the ‘simple’ tracker for cross-frame identity matching (see https://sleap.ai/guides/proofreading.html#tracking-method-details for more details). For each video analysis we exported both the user labels and the predicted labels.

### Analysis

Here we detail three types of analyses we performed in order to assess the performance of SLEAP in the case of plant growth dynamics: training time, prediction accuracy, and comparison to other tracking methods. All statistical tests were calculated to a significance level of 0.05.

### Training time

To examine whether training time was influenced by the total number of labels per frame and the file size of each video, since different video analyses converged after a different number of epochs, we first calculated the mean training time SLEAP needed per epoch. We then calculated a linear mixed model (LMER) [21] with the mean training time per epoch as response variable, the number of labelled points per frame as well as the file size of the input video as explanatory variables. Because we had repeated analyses for each video differing only in the number of labelled training frames, we added this number as a random term in the model.

### Predictive power

To determine the predictive power of the trained models we calculated the percentage of frames in which the labelled points and the number of individuals were correctly predicted. For this and the following analyses we categorized the labelled points for each video analysis into categories marked as B (representing base points), M (midpoints), T (tips) and TB (branching points). For more detailed definitions according to the observed organ, see Table 2, and Fig. 2f for an example.

**Table 2:** Overview over node-label categories for each video analysis

Label category	Video analysis	Description
B	AG	Root tip
	AP, BT & SP	Base of the plant
	STL	Base of the leaf
	STP	Centre of the plant crown
M	AG, AP, BT, SP, STL & STP	Midpoint(s) along the stem or leaf
TB	AG, AP, SP	Branching of the stem towards the leaves
T	AG, AP, BT, SP, STL & STP	Leaf tips

For each plant part across each video analysis we used Kruskal–Wallis tests [22] to check whether the percentage of frames in which a plant part was identified depended on the number of user-labelled frames used to train a model. In some instances, such an analysis was not possible, for example, when SLEAP was able to correctly identify all plant parts across 100% of frames. In a next step, we calculated the Euclidean distance between each user labelled point and the corresponding predicted point for each of the 20 user labelled frames and each video analyses. To test whether the number of labelled training frames as well as the plant part had an influence on prediction accuracy, we then calculated separate linear models for each video analysis [23, 24] with the logarithm of the distance between predicted and user-labelled points as response variable and the interaction between plant part category and the number of labelled training frames as explanatory variables. Finally, we normalized the distances by the width of each image to make them comparable across video analyses and compared the median normalized distance and the overall normalized distance across video analyses using a Kruskal–Wallis test, as assumptions for a parametric test were violated.

### Comparison with RootStem Extractor

SLEAP predicts the image coordinates of morphological landmarks but not derived features like shoot angles. To evaluate its performance for downstream analysis tasks, we next compared it to a specialized algorithm designed for plant phenotyping. To do this, we calculated and plotted the angles of the shoot tip for the AG, AP and SP analysis to demonstrate that the information provided by SLEAP can be used to investigate tropisms. Specifically, we calculated shoot angles, as the angle between the highest midpoint along the shoot and the tip branch (i.e., from 5–6 for AG, 2–3 for AP and 3–4 for SP). We then compared the tip angles of the AP video analysis calculated with SLEAP with those calculated using the RootStem Extractor (Chauvet et al., 2016; https://forgemia.inra.fr/hugo.chauvet-thiry/rootstemextractor) and kindly provided by M. Rivière. Angles in RootStem Extractor are averaged along the top part of the shoot, equal to two mean diameters of the shoot [12]. As a similar proof of concept, we plotted the nastic circumnutation movements for the sunflower plants (STP).

## Results

We successfully tracked the movement of plants across all videos, and the models successfully predicted individuals on all frames for each of the different video analyses. See Additional file 1: Video S1; Additional file 2: Video S2; Additional file 3: Video S3; Additional file 4: Video S4; Additional file 5: Video S5; Additional file 6: Video S6 and Fig. 2 for an overview of the total movement trajectories of plants using the models trained with 20 user labelled frames, as well as an example skeleton for each video-analysis. Training time increased with both the number of points to track on each frame, and with the file size of the input video (see Additional file 7: Table S1, Figure S1). For each of the video analyses with a side view (AG, AP, BT and SP), we predicted the correct number of instances on all frames. For the video analyses tracking sunflower crowns from the top, the models predicted all instances correctly across frames for the STP video analysis (tracking individual plants). However, in the STL analysis (tracking individual leaves) only 28.6% of instances were correctly identified on all frames across all three models (i.e., 5, 10 or 20 user-labelled frames). When comparing the different models, we found that the model trained on 10 user-labelled frames outperformed the other two. Specifically, this model found on average 14 out of 21 individuals compared to 12 for the model trained on 20 user-labelled frames, and 11 for the model trained on 5 user-labelled frames (Additional file 7: Figure S2, Table S2). We note that while both approaches (STP and STL) are able to track the same points, treating whole plants as instances greatly improves reliability of tracking, likely owing to the heavy occlusion across leaves in the STL approach.

**Fig. 3:**
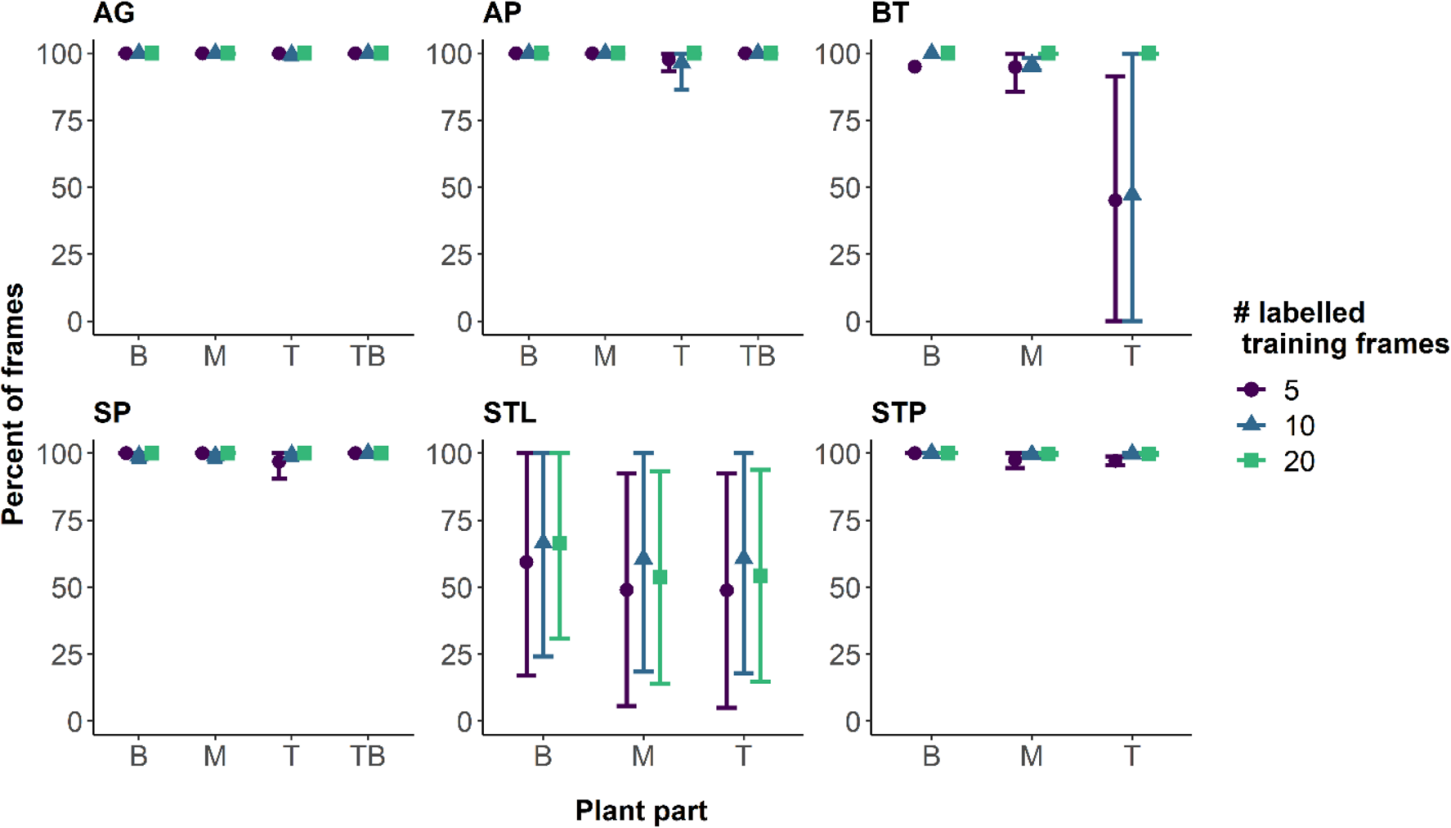
For each video analysis we plotted the percentage of frames for which a specific labelled plant part was identified, where B (base), M (mid-section), T (tip) and TB (tip branching) represent the label categories detailed in Table 2. Plotted are the mean and standard error (calculated across all individuals in a video). Different colours and shapes represent the number of frames used to train each model

The correct prediction of the presence of a plant part across frames did not depend on the number of user-labelled frames used for training for any video analysis (Additional file 7: Table S3, Fig. 3), apart from the STP video analysis where we found a trend that correct predictions of leaf tips increased with the number of training user-labelled frames.

**Fig. 4:**
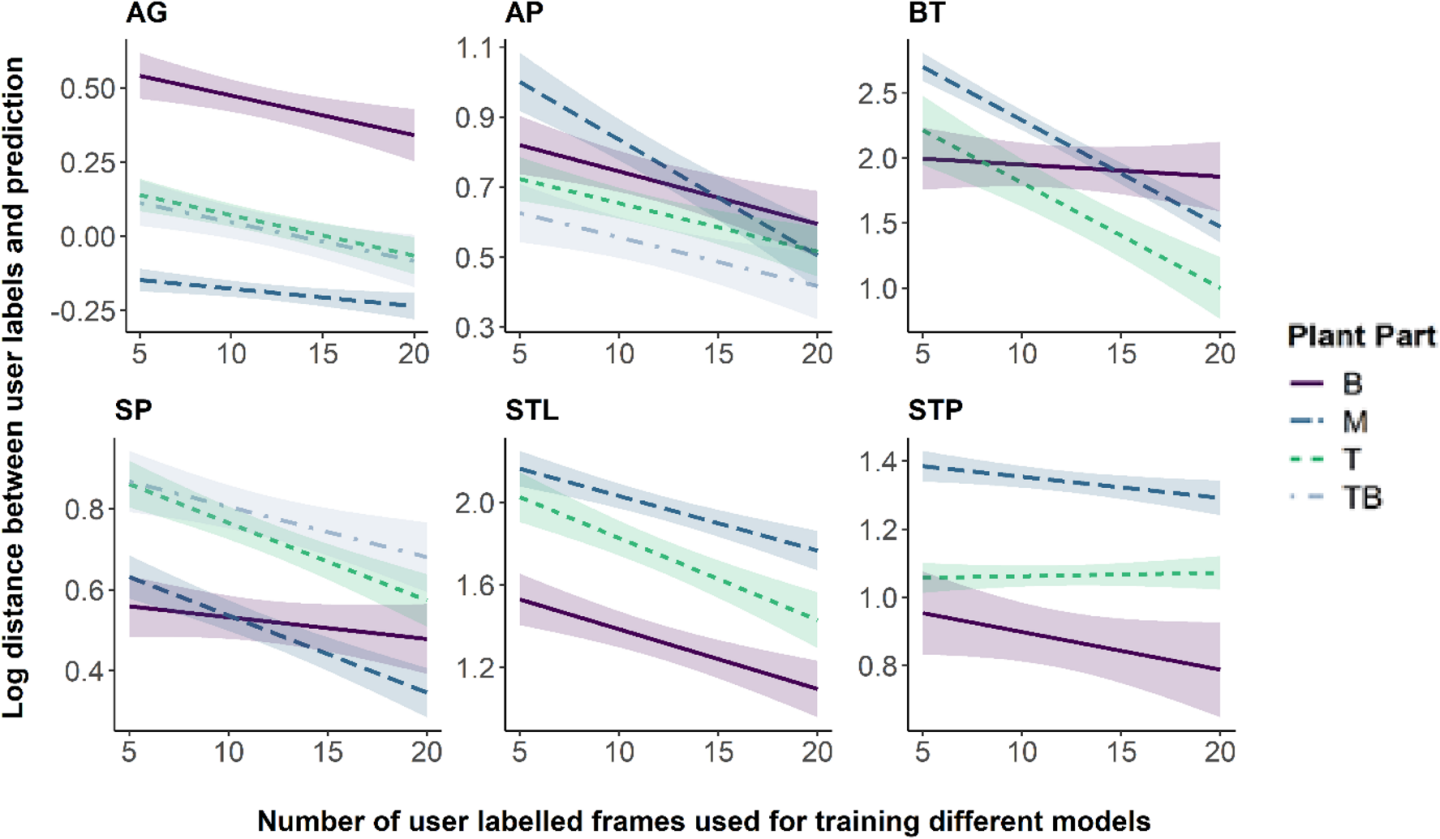
Plotted are the results of a linear model (estimate and 95% confidence bands) with the logarithm of the distance between user labelled points and predicted points as a response variable and the interaction of plant part and the number of labelled training frames the models as explanatory variables. For this analysis plant parts were categorized to make them comparable across the different video analysis, where B (base), M (mid-section), T (tip) and TB (tip branching) represent the label categories detailed in Table 2. Note that due to the different image sizes across videos, distances cannot be directly compared across analyses in this plot

The prediction accuracy, measured as distance between predicted and user-labelled plant parts, was overall high, but was explained by different factors across the different video analyses (Additional file 7: Tables S4–S9). For the AG video analysis prediction accuracy was significantly lower for the root tip (B) compared to the mid points (M) along the root and shoot, the tip (T) and tip branch (TB). There was also a significant effect of the number of labelled training frames with more labelled training frames resulting in more accurate predictions independent of the plant part being tracked (Fig. 4, Table S4). In the AP video analysis accuracy was significantly lower for the mid points (M) compared to the base of the shoot (B). We found no difference in accuracy between the shoot base, the tip (T) and tip branch (TB). Again, the number of user labelled frames used to train the different models had a significant effect on accuracy, with more labelled frames used for training leading to more accurate results independent of the plant part being tracked (Fig. 4, Additional file 7: Table S5). For the BT video analysis, we found a significant interaction between the plant part and the number of labelled training frames. Specifically, accuracy increased significantly with increasing number of labelled training frames for the mid points along the shoot (M) and the tip (T) (Fig. 4, Additional file 7: Table S6). For the SP video analysis, we found no effect of the number of labelled training frames. There was also no difference in the accuracy between the mid points along the shoot (M) and the base of the shoot (B), though accuracy was significantly lower for the tip (T) and the tip branch (TB) than for the shoot base (Fig. 4, Additional file 7: Table S7). The accuracy in the STL video analysis was highest for the base of each leaf (B) compared to the midpoint along a leaf (M) and the leaf tip (T) and increased with the number of labelled training frames (Fig. 4, Additional file 7: Table S8). For the STP video analysis accuracy did not depend on the number of labelled training frames, though the midpoint along each leaf (M) was less accurately tracked than the leaf tip (T) or centre of the plant (B) (Fig. 4, Additional file 7: Table S9).

Median normalized prediction accuracy was calculated in order to compare accuracy across video analyses, measured as the normalized distance between user labelled and predicted plant parts. This differed significantly across the different video analyses (χ^2^ = 1536, df = 5, p-value < 0.001, Additional file 7: Figure S3a), consistent with the overall differences in the distance distributions. Some video analyses are characterized by wider distributions, i.e., STL (χ^2^ = 13.09, df = 5, p-value = 0.023, Additional file 7: Figure S3b). In an additional step, we checked whether models trained on one video of an experimental setup can be used to track plants in a different video of the same. However, this did not work for any video analysis presented here. We note that the short labelling and training times still make it feasible to track new sessions in a reasonable amount of time, but future work using more and more diverse labels may improve the ability of models to generalize to new sessions.

**Fig. 5:**
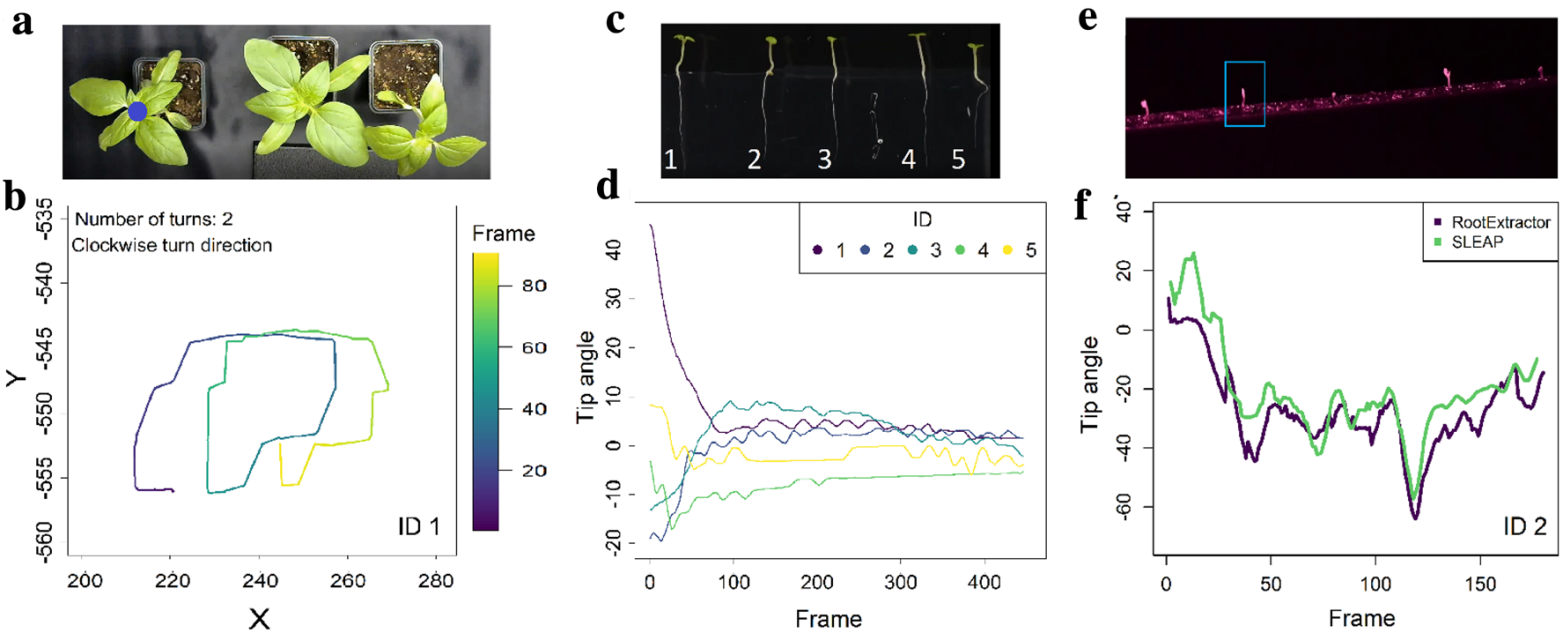
**a** Sunflower shade avoidance setup. The blue dot on the first plant on the left represents the centre of the plant. The trajectory of this point (circumnutations)—a clockwise motion—as predicted by the model trained on 20 user labelled frames is shown in **b**. **c** *Arabidopsis* Gravitropism setup with the numbers representing the individuals as plotted in **d**. In **d** are plotted the tip angle of the shoots of the AG video analysis for each of the individuals in the video for the models trained on 20 user labelled frames. **e** *Arabidopsis* Phototropism setup, the blue square marks the plant whose shoot angles are tracked in **f**. **f** Comparison of the shoot angles for ID 2 in the AP video analysis across frames, extracted using RootStem Extractor (dark purple line) and using SLEAP from the of the model trained on 20 user labelled frames (green line). Please note that angles were calculated between point 2 and 3 for SLEAP (see Fig. 2) but were averaged along the whole upper shoot for Root Extractor. We used a rolling mean across 5 frames to smooth the angles calculated from SLEAP both in **d** and **f**

Figure 5 shows different types of data that we extracted from the videos, including the periodic dynamics of circumnutations of sunflowers (STP), and the gravitropic and phototropic responses of *Arabidopsis* seedlings (AG and AP). Additional tropic responses of plant shoots extracted from AG, AP and SP video analysis can be found in Additional file 7: Figure S4. When comparing the shoot angles for the AP video analysis between different extraction methods (RootStem Extractor vs. SLEAP), we found good overall agreement between the two methods (Fig. 5f). It is important to note, that the two programs calculate angles differently. RootStem Extractor averages angles along the upper shoot (with one point ~ every 0.3 pixels) and the angles from SLEAP are calculated between point 2 and 3 on the skeleton (see Fig. 2). Accordingly, the angles are unlikely to match exactly but if the two programs track plants similarly well, the overall change in angle should be similar.

## Discussion

In this work we adopted Social LEAP Estimates Animal Poses (SLEAP)—a tool based on convolutional neural networks originally aimed at estimating animal poses—for plant tracking. We tested it on time-lapse videos of a variety of cases including: (i) organ types and imaging angles (e.g. top-view crown leaves and side-view shoots and roots), (ii) lighting conditions (full spectrum, blue light, IR), (iii) plant morphologies and scales (100 μm-scale *Arabidopsis* seedlings and cm-scale sunflowers and beans), and (iv) movement types (circumnutations, tropisms and twining). SLEAP successfully identified individuals and labelled points and tracked their movement across all types of the videos tested here. Training time depended both on the number of labelled points per frame as well as the size of the input video. We note that the exact training time will depend on the machine used and will be quicker using a computer with an integrated GPU.

The top view of plants provides a number of challenges compared to the side view. Particularly, plant crowns are comprised of many ‘sub-individuals’ (leaves) which drastically change their shape during growth (as opposed to the one-dimensional growth of a single stem) and can furthermore occlude each other. We found that tracking the whole plant rather than single leaves proved more effective, by allowing tracking of all instances in all frames with consistently higher accuracy. Tracking single leaves has the benefit of a simple skeleton which remains fixed over time, in contrast to whole plants where new leaves can grow at any moment, requiring predefinition of more nodes within the skeleton than might be present for most of a video. However, though the skeleton was less complex (only 4 points rather than 19), there were many more instances to track. In many cases specific plant parts were not assigned to the correct instance, thus leading to fewer instances identified than were present. For instance, too few instances were found when two or more leaves were partly occluded, and the remaining visible parts were assigned to a single instance. The STP analysis handled occlusions better, even when models were trained on only a few user-labelled frames.

The correct prediction of specific plant parts did not depend on the number of labelled training frames for any video analyses, aside from the STP analysis, where there was a small trend that predictions of leaf tips increased with the number of training user-labelled frames. Overall, when plant parts were found, accuracy was high, though the distance between predicted and user labelled points depended on the specific plant part, and for some video analysis also on the number of user labelled frames. Accuracy in our analysis seemed to depend on how well features could be distinguished. For instance, in the AP analysis where images are rather noisy, prediction accuracy was lower for the points along the shoot, where there are no obvious distinguishing features, compared to the branching point, the leaf tips or the base of the plant. Similarly, the root tip in the AG video is very faint, and while the prediction was still very accurate, it was less accurate for this plant part compared to the other parts. This contrasts with the SP analysis where painted dots along otherwise featureless stems of sunflower seedlings served as tracking aid, while for STL and STP, the veins in the leaves offered good natural distinguishing features. For most of our analyses, accuracy increased with the number of labelled training frames, indicating that more is better. However, for some plant parts there was no difference in accuracy based on the number of user labelled frames, suggesting that there is an optimal number of frames after which any additional labelling will not lead to a further increase in accuracy.

For optimal position tracking, a trained model would ideally be transferred from one video to another of the same experiment type, thus reducing labelling and training time. However, we found that our models tended to overfit, tracking the specific features of each individual/plant part and thus not allowing to transfer trained models from one video to another. We note that the short labelling and training times still make it feasible to track new sessions in a reasonable amount of time, but future work using more and more diverse labels may improve the ability of models to generalize to new sessions. Occlusions can provide another important challenge when trying to track the position and movement of plant parts. We found that connecting all nodes to the base (centre of the crown) helped deal with possible occlusions of the middle of a leaf. Similarly, such skeletons also help to track the twining tip of the bean plant in the BT video analysis when part of the stem was hidden behind the support (e.g. we added branching connections from point along the stem to all the following points (1–2, 1–3, 1–4), rather than connecting these points linearly along the stem (1–2, 2–3, 3–4)).

Importantly, as the program was not built for plants whose structures change with growth, the skeleton needs to be carefully considered. High image quality with clearly separate individuals allows for high prediction accuracy with only 5 labelled frames per video. When image quality is low, additional markers on the plants can help to improve predictions. Top-view images are most challenging, since leaves change their morphology in 2D, and occlude each other often. Here different skeleton definitions help improve tracking.

## Conclusion

SLEAP offers a useful tool for fast and high throughput tracking of plant movement in time-lapse videos. We were able to use SLEAP to extract information on a variety of dynamical growth-driven plant processes, including plant tropisms, circumnutations movements and even interactions, such as dynamic leaf. We find that these results encourage the use of SLEAP, or of tools based on convolutional neural networks in general, on plants. Further development of such tools will optimize their success on such occluding structures of changing morphologies and may prove useful in other fields as well.

## Supplementary Information

**Additional file 1: Video S1. **Arabidopsis gravitropism (AG), corresponding to Fig. 2a.

**Additional file 2: Video S2. **Arabidopsis phototropism (AP), corresponding to Fig. 2b.

**Additional file 3: Video S3. **Bean twining (BT), corresponding to Fig. 2c.

**Additional file 4: Video S4.** Sunflower phototropism (SP), corresponding to Fig. 2d.

**Additional file 5: Video S5.** Sunflower shade avoidance, tracking individual leaves (STL), corresponding to Fig. 2e.

**Additional file 6: Video S6. **Sunflower shade avoidance, tracking individual plants (STP), corresponding to Fig. 2f.

**Additional file 7.** Additional figures S1–S5 and tables S1–S9.

## Data Availability

The datasets during and/or analysed during the current study available at: https://zenodo.org/record/5764169#.YbCK0_FBxqt, https://doi.org/10.5281/zenodo.5764169, which includes: (1) raw videos of the timelapse for each analysis. (2) The.slp files for each video analysis, which can be loaded into SLEAP and contain the 5, 10 or 20 labelled training frames. From these files users can train the models either directly in the GUI or on Google CoLab. (3) The output analysis file with only the user labelled training frames [20]. These can be also directly generated from the .slp files. (4) The output analysis files with the predicted data for each video analysis.
