# Supplementary material for: Fast estimation of plant growth dynamics using deep neural networks
Source: Plant Methods. 2022 Feb 20;18:21. doi: 10.1186/s13007-022-00851-9 (PMC8858456; doi:10.1186/s13007-022-00851-9)
Supplement: Supplementary file 7 — Additional file 7. Additional figures S1–S5 and tables S1–S9. [file 13007_2022_851_MOESM7_ESM.docx]

**Additional Material**

Table S 1: Results of the linear mixed model with the logarithm of training time as response variable and the number of labelled points per frame as well as the size of the input video as explanatory variable. We controlled for the number of labelled frames for the training in the random term.

| Explanatory Variable | Estimate | Std. Error | df | t value | p value |
| --- | --- | --- | --- | --- | --- |
| Intercept | -0.325 | 0.243 | 6.922 | -1.339 | 0.222 |
| Number of labelled points per frame | 0.021 | 0.004 | 14.427 | 4.9757 | <0.001 |
| Video size | 0.026 | 0.006 | 3.460 | 4.678 | 0.013 |


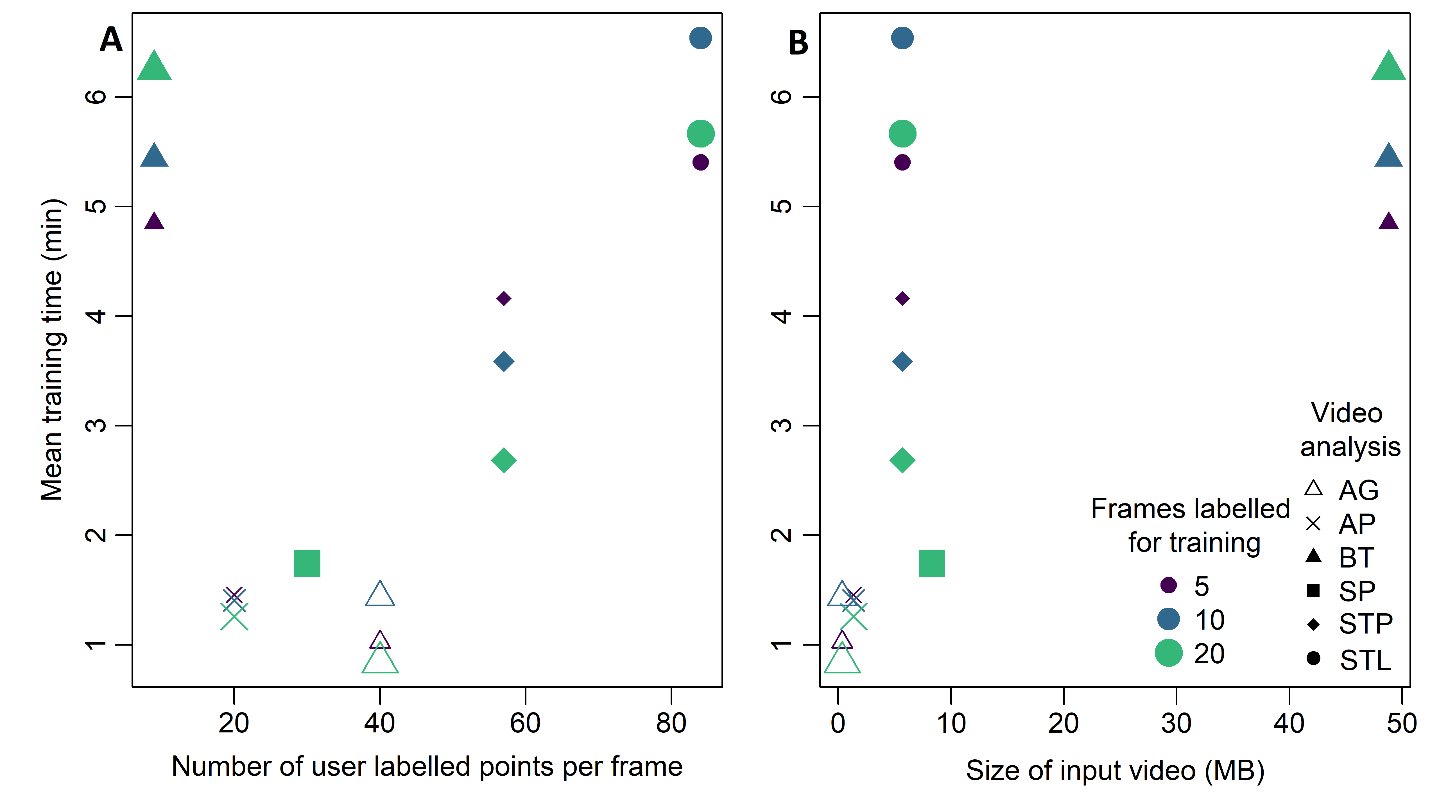


Figure S1: The mean training time needed for each epoch depending on **A** the number of user labelled points per frame and **B** the size of the input video in MB. Plotted is the raw data.

Table S 2: Results from a linear model comparing the difference in the number of individuals found (in the predicted vs the user labelled frames for the STL video analysis) across the different models which were predicted on 5 (intercept), 10 or 20 user labelled frames.

| Explanatory variable | Estimate | Std. Error | | t value | | l CI | | u CI | | p value | |
| --- | --- | --- | --- | --- | --- | --- | --- | --- | --- | --- | --- |
| Intercept | 9.85 | 0.237 | 41.588 | | 9.376 | | -2.721 | | <0.001 | |  |
| 10 Labels | -2.05 | 0.335 | -6.12 | | 9.376 | | -2.721 | | <0.001 | |  |
| 20 Labels | -0.55 | 0.335 | -1.642 | | 9.376 | | -2.721 | | 0.106 | |  |


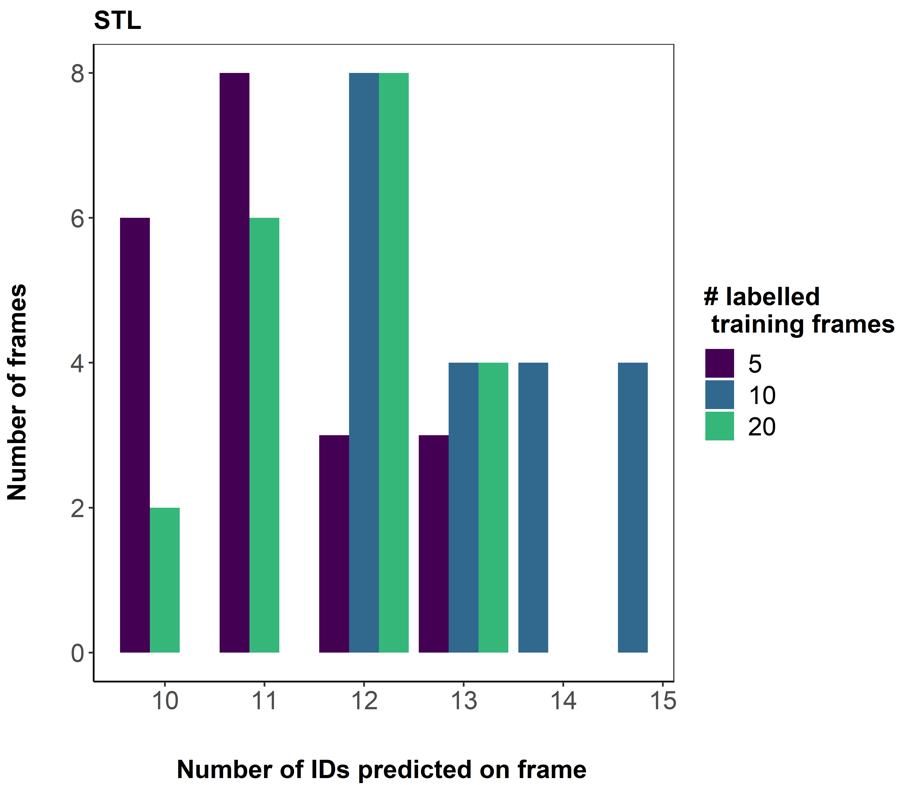


Figure S2: Plotted is a histogram of the number of individuals predicted by each of the trained models (trained on 5, 10 or 20 user labelled frames) per frame for the STL video analysis. In the video were a total of 19 individuals (here indicating the separate leaves) per frame, though none of the models managed to predict all 19 correctly.

Table S 3: Results from Kruskal-Wallis tests to compare whether the percent of plant parts found across frames differed according to predictions using 5, 10 or 20 user labelled frames for training. In some instances, we could not compute a Kruskal-Wallis test as all percentages were equal. In this case we indicated the range of plant parts for which this was the case and entered a NA in the table. Significant results are marked in bold.

| Experiment | Plant part | χ^2^ | DF | p-value |
| --- | --- | --- | --- | --- |
| AG | B | NA | 2 | NA |
| AG | M | NA | 2 | NA |
| AG | TB | NA | 2 | NA |
| AG | T | 2.00 | 2 | 0.368 |
| AP | B | NA | 2 | NA |
| AP | M | NA | 2 | NA |
| AP | TB | NA | 2 | NA |
| AP | T | 2.013 | 2 | 0.366 |
| BT | B | 2 | 2 | 0.368 |
| BT | M | 5.333 | 2 | 0.069 |
| BT | T | 3.529 | 2 | 0.171 |
| SP | B | 2 | 2 | 0.368 |
| SP | M | 4.143 | 2 | 0.126 |
| SP | TB | NA | 2 | NA |
| SP | T | 2.330 | 2 | 0.312 |
| STL | B | 0.087 | 2 | 0.958 |
| STL | M | 0.404 | 2 | 0.817 |
| STL | T | 0.173 | 2 | 0.917 |
| STP | B | NA | 2 | NA |
| STP | M | 1.697 | 2 | 0.428 |
| STP | T | 5.915 | 2 | 0.052 |

Table S 4: Results from a linear model for the *Arabidopsis* gravitropism data (AG) with the logarithm of the distance between user labels and predicted labels per body section as response variable and the plant section (Base, Mid-section, Tip branching and Tip) and the number of frames labelled for training of the model (NLabels) as explanatory variables. Significant results are marked in bold.

| Explanatory Variables | Estimate | l-95% CI | u-95% CI | Std. Error | t value | p-value |
| --- | --- | --- | --- | --- | --- | --- |
| **Intercept** | **0.608** | **0.437** | **0.779** | **0.087** | **6.969** | **<0.001** |
| **Plant part: Mid** | **-0.725** | **-0.917** | **-0.534** | **0.098** | **-7.439** | **<0.001** |
| **Plant part: Tip** | **-0.400** | **-0.610** | **-0.191** | **0.107** | **-3.744** | **<0.001** |
| **Plant part: Tip branch** | **-0.430** | **-0.672** | **-0.188** | **0.123** | **-3.487** | **<0.001** |
| **NLabels** | **-0.013** | **-0.026** | **0.000** | **0.007** | **-2.022** | **0.043** |
| Mid x NLabels | 0.007 | -0.007 | 0.022 | 0.007 | 1.011 | 0.312 |
| T x NLabels | 0.000 | -0.016 | 0.016 | 0.008 | -0.037 | 0.97 |
| TB x NLabels | 0.000 | -0.018 | 0.019 | 0.009 | 0.033 | 0.973 |

Table S 5: Results from a linear model for the *Arabidopsis* phototropism data (AP) with the distance between user labels and predicted labels per body section as response variable and the plant section (Base, Mid-section, Tip branching and Tip) and the number of frames labelled for training of the model (NLabels) as explanatory variables. Significant results are marked in bold.

| Explanatory Variables | Estimate | l-95% CI | u-95% CI | Std. Error | t value | p-value |
| --- | --- | --- | --- | --- | --- | --- |
| **Intercept** | **0.895** | **0.711** | **1.079** | **0.094** | **9.548** | **<0.001** |
| **Plant part: Mid** | **0.270** | **0.010** | **0.530** | **0.133** | **2.038** | **0.042** |
| Plant part: Tip | -0.103 | -0.333 | 0.128 | 0.117 | -0.876 | 0.381 |
| Plant part: Tip branch | -0.200 | -0.460 | 0.060 | 0.133 | -1.508 | 0.132 |
| **NLabels** | **-0.015** | **-0.029** | **-0.001** | **0.007** | **-2.119** | **0.034** |
| Mid x NLabels | -0.018 | -0.038 | 0.002 | 0.010 | -1.797 | 0.073 |
| T x NLabels | 0.001 | -0.016 | 0.019 | 0.009 | 0.137 | 0.891 |
| TB x NLabels | 0.001 | -0.019 | 0.021 | 0.010 | 0.115 | 0.909 |

Table S 6: Results from a linear model for the Bean twining data (BT) with the distance between user labels and predicted labels per body section as response variable and the plant section (Base, Mid-section, Tip branching and Tip) and the number of frames labelled for training of the model as explanatory variables. Significant results are marked in bold.

| Explanatory Variables | Estimate | l-95% CI | u-95% CI | Std. Error | t value | p-value |
| --- | --- | --- | --- | --- | --- | --- |
| **Intercept** | **2.042** | **1.511** | **2.573** | **0.270** | **7.557** | **<0.001** |
| **Plant part: Mid** | **1.068** | **0.485** | **1.651** | **0.296** | **3.603** | **<0.001** |
| Plant part: Tip | 0.577 | -0.205 | 1.358 | 0.397 | 1.451 | 0.148 |
| NLabels | -0.009 | -0.049 | 0.031 | 0.02 | -0.456 | 0.649 |
| **Mid x NLabels** | **-0.073** | **-0.116** | **-0.029** | **0.022** | **-3.271** | **0.001** |
| **Tip x NLabels** | **-0.072** | **-0.128** | **-0.016** | **0.028** | **-2.526** | **0.012** |

Table S 7: Results from a linear model for the Sunflower phototropism data (SP) with the distance between user labels and predicted labels per body section as response variable and the plant section (Base, Mid-section, Tip branching and Tip) and the number of frames labelled for training of the model (NLabels) as explanatory variables. Significant results are marked in bold.

| Explanatory Variables | Estimate | l-95% CI | u-95% CI | Std. Error | t value | p-value |
| --- | --- | --- | --- | --- | --- | --- |
| **Intercept** | **0.586** | **0.419** | **0.754** | **0.085** | **6.863** | **<0.001** |
| Plant part: Mid | 0.141 | -0.064 | 0.346 | 0.105 | 1.346 | 0.178 |
| **Plant part: Tip** | **0.371** | **0.161** | **0.582** | **0.107** | **3.458** | **0.001** |
| **Plant part: Tip branch** | **0.344** | **0.107** | **0.581** | **0.121** | **2.850** | **0.004** |
| NLabels | -0.005 | -0.018 | 0.007 | 0.006 | -0.833 | 0.405 |
| Mid x NLabels | -0.014 | -0.029 | 0.002 | 0.008 | -1.730 | 0.084 |
| T x NLabels | -0.014 | -0.03 | 0.002 | 0.008 | -1.705 | 0.088 |
| TB x NLabels | -0.007 | -0.025 | 0.011 | 0.009 | -0.778 | 0.436 |

Table S 8: Results from a linear model for the Sunflower shading leaf data (STL) with the distance between user labels and predicted labels per body section as response variable and the plant section (Base, Mid-section, Tip branching and Tip) and the number of frames labelled for training of the model (NLabels) as explanatory variables. Significant results are marked in bold.

| Explanatory Variables | Estimate | l-95% CI | u-95% CI | Std. Error | t value | p-value |
| --- | --- | --- | --- | --- | --- | --- |
| **Intercept** | **1.674** | **1.398** | **1.950** | **0.141** | **11.890** | **<0.001** |
| **Plant part: Mid** | **0.621** | **0.285** | **0.957** | **0.171** | **3.626** | **<0.001** |
| **Plant part: Tip** | **0.550** | **0.164** | **0.936** | **0.197** | **2.795** | **0.005** |
| **NLabels** | **-0.029** | **-0.049** | **-0.008** | **0.010** | **-2.761** | **0.006** |
| Mid x NLabels | 0.002 | -0.023 | 0.028 | 0.013 | 0.192 | 0.847 |
| Tip x NLabels | -0.011 | -0.040 | 0.018 | 0.015 | -0.735 | 0.462 |

Table S 9: Results from a linear model for the Sunflower shading full plant data (STP) with the distance between user labels and predicted labels per body section as response variable and the plant section (Base, Mid-section, Tip branching and Tip) and the number of frames labelled for training of the model (NLabels) as explanatory variables. Significant results are marked in bold.

| Explanatory Variables | Estimate | l-95% CI | u-95% CI | Std. Error | t value | p-value |
| --- | --- | --- | --- | --- | --- | --- |
| **Intercept** | **1.010** | **0.739** | **1.280** | **0.138** | **7.317** | **<0.001** |
| **Plant part: Mid** | **0.406** | **0.118** | **0.694** | **0.147** | **2.763** | **0.006** |
| Plant part: Tip | 0.043 | -0.244 | 0.33 | 0.146 | 0.293 | 0.769 |
| NLabels | -0.011 | -0.032 | 0.009 | 0.010 | -1.062 | 0.288 |
| Mid x NLabels | 0.005 | -0.017 | 0.027 | 0.011 | 0.440 | 0.660 |
| Tip x NLabels | 0.012 | -0.010 | 0.034 | 0.011 | 1.090 | 0.276 |


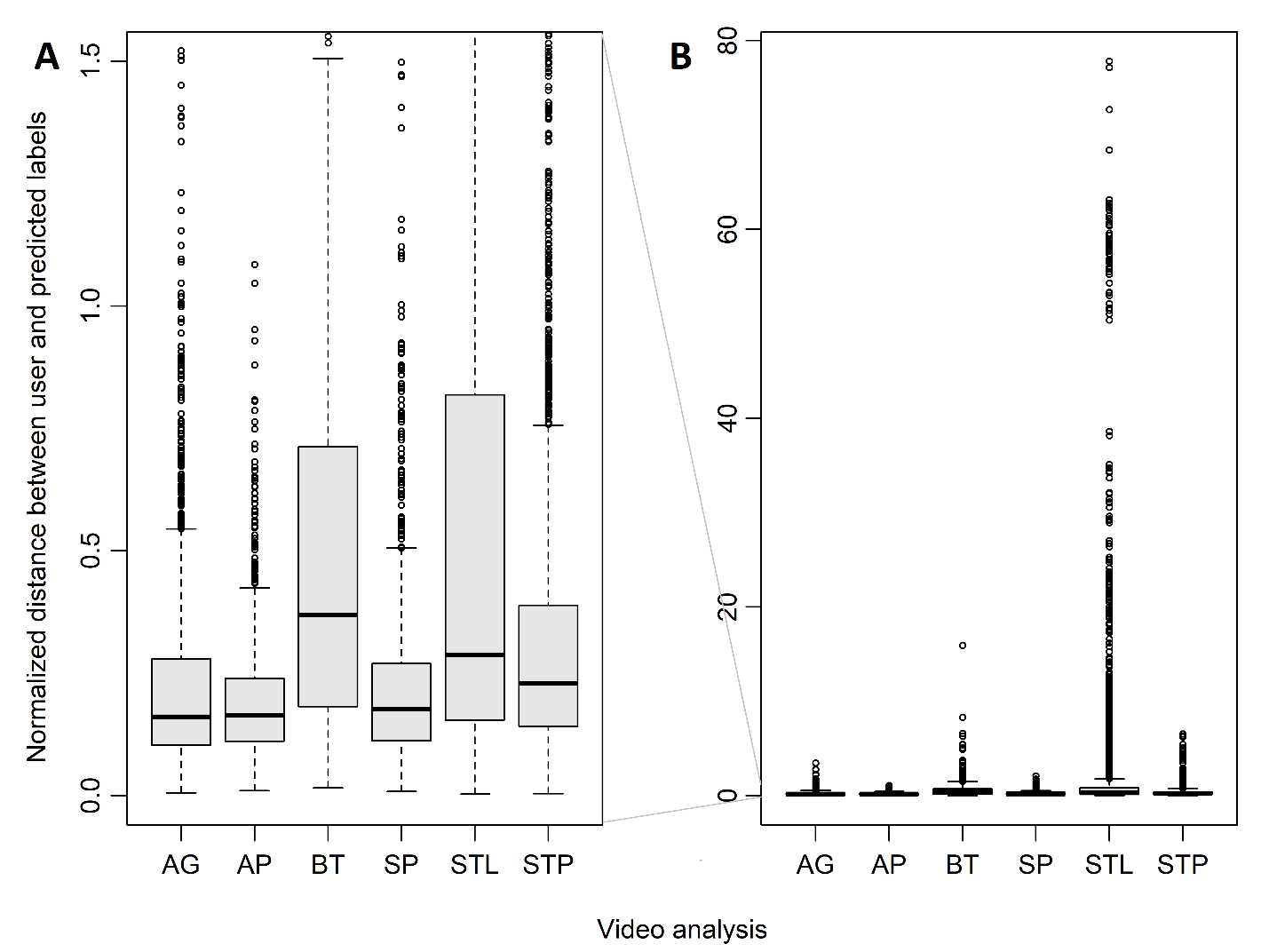


Figure S3: Boxplots of the normalized distance between user- and predicted labels across all video analyses. Distances were normalized by the image width of each video analysis. **A** represents the zoomed in version of **B**. The thick black line represents the median, boxes the 25% to 75% quantiles or interquartile range (IQR), the whiskers 1.5*IQR below and above the box and the remaining points all remaining values.


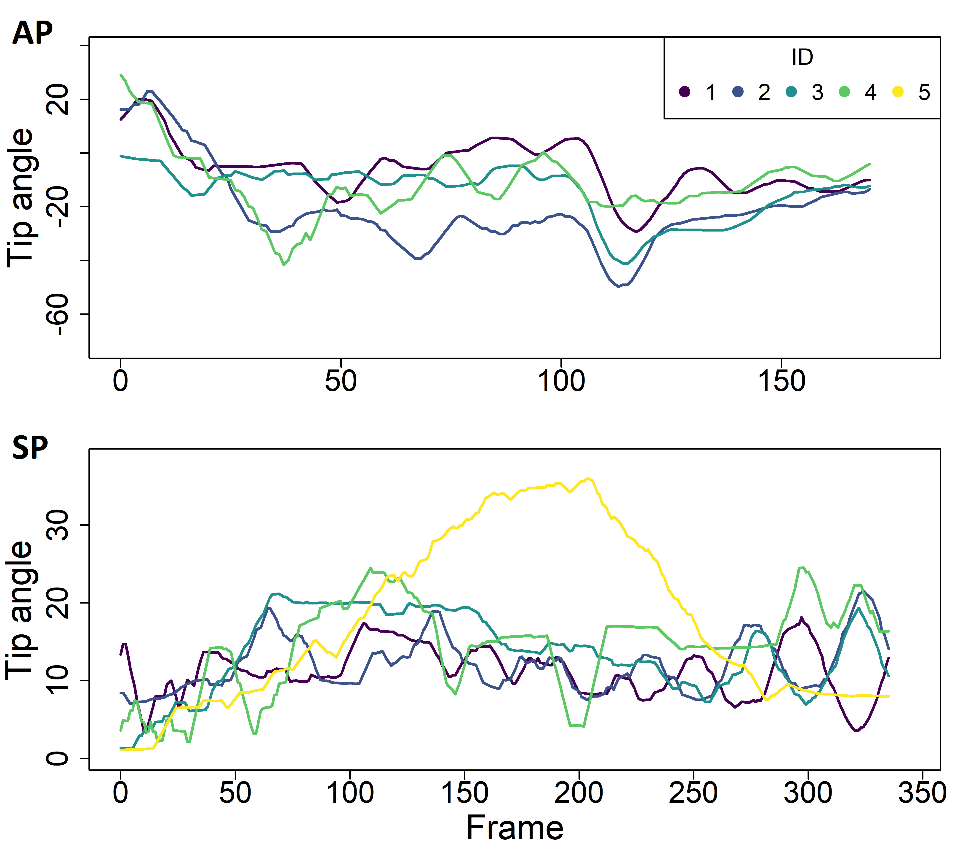


Figure S 4: Example of the tip angle of the shoots of the AP and SP video analysis for each of the individuals in the video (individuals were counted from left to right) for the models trained on 20 user labelled frames. For each of the video analysis we calculated the angle from the highest midpoint along the shoot to the tip branch (see Figure 2 for details of each skeleton). We used a rolling mean across 10 frames to smooth the angles.


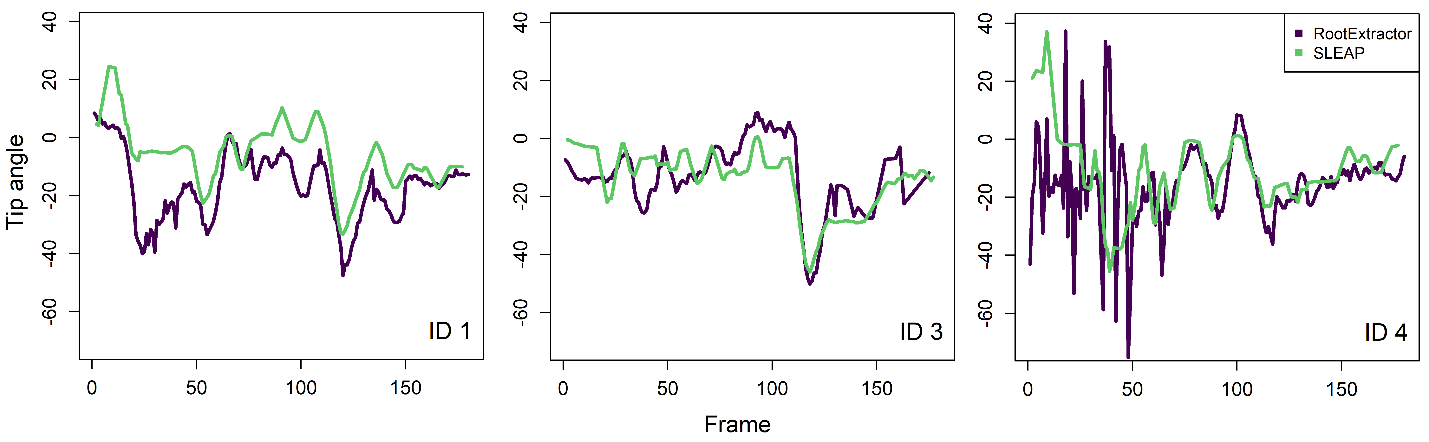


Figure S 5: Comparison between the shoot angles for the first, third and fourth plant in the AP video analysis across frames, extracted using RootStem Extractor (dark purple line) and angles extracted using SLEAP from the of the model trained on 20 user labelled frames (green line). We used a rolling mean across 5 frames to smooth the angles calculated from SLEAP. Please note that angles were calculated between point 2 and 3 for SLEAP (see Figure 2) but where averaged along the whole upper shoot for Root Extractor. See Figure 5 c) for a comparison for ID 2.

Additional videos

For each video analysis we added a video with the animated predicted tracks as outputted directly from SLEAP. For these videos we faded from the original input video to the video with movement tracks using Adobe Premier Pro. They are named by the video analysis, the number of training frames and in the case of the Sunflower shade avoidance video whether we tracked leaves or plants, i.e.:

- Arabidopsis_Gravitropism_20Labels.avi,
- Arabidopsis_Phototropism_20Labels.avi,
- Bean_Twining_20Labels.avi,
- Sunflower_Phototropism_20Labels.avi,
- SunflowerShadeAvoidance_20Labels_Leaf.avi
- SunflowerShadeAvoidance_20Labels_Plant.avi.

The video for the *Arabidopis* gravitropism was rendered at 30 frames per second, the Arabidopsis phototropism, bean twining, and sunflower phototropism at 15 frames a second, and the sunflower shade avoidance videos at 24 frames a second.
